# A hybrid model for youth engagement in adolescent health studies: insights from the teen research advisors program at a Midwestern pediatric Academic Medical Center in the United States

**DOI:** 10.3389/fpubh.2025.1668387

**Published:** 2025-10-21

**Authors:** John Tumberger, Anna E. Burns, Michael Bartkoski, Mariah Brewe, Amelia Whittier, Kendyll Gethers, Ailisi Bogdon, Emerie Peterson, Stephani L. Stancil

**Affiliations:** ^1^Division of Clinical Pharmacology, Toxicology and Therapeutic Innovation, Children’s Mercy Kansas City, Kansas City, MO, United States; ^2^Department of Pediatrics, Division of Adolescent and Young Adult Medicine, Children’s Mercy Kansas City, Kansas City, MO, United States; ^3^University of Kansas School of Medicine, Kansas City, KS, United States; ^4^Department of Pediatrics, University of Missouri Kansas City School of Medicine and University of Kansas School of Medicine, Kansas City, MO, United States

**Keywords:** adolescent, research, community engagement, bidirectional impact, hybrid participation model

## Abstract

**Background:**

Engaging adolescents in research ensures studies are relevant, ethical, and beneficial while fostering authentic and applicable findings. Traditional in-person advisory boards face barriers to equitable participation, highlighting the need for innovative, flexible models tailored to specific research programs and individuals with lived experience. The primary aim of this article is to describe a novel hybrid youth advisory board suitable for informing ongoing operations of a research program while supporting youths’ education and career exploration. Our secondary aim was to evaluate initial impact over the first 2 years of the program (2023–2024).

**Methods:**

Adolescents aged 12–21 with prior involvement in mental health research at Children’s Mercy Kansas City (Kansas City, United States) were invited to join a hybrid teen advisory board. The advisory board structure and priorities were continuously shaped by youth. Participation included monthly discussion boards, quarterly huddles, enrichment events, and one-on-one mentorship for personal and professional development. Through a mixed-methods approach, initial program evaluation assessed alignment with the Advisor-defined objectives, program engagement, and bidirectional impact through thematic qualitative analysis and quantitative metrics.

**Results:**

During the first 2 years, 11 youth (aged 13–20 years) participated as Teen Research Advisors (TRA) for an average of 12 ± 8 months. For any given monthly online, asynchronous discussion (*n* = 23 discussions), >80% of TRA contributed comments and peer responses. Quarterly Huddles (*n* = 7 huddles) were attended by 70% of TRAs and in-person enrichment events (*n* = 4 events) received positive feedback (“very helpful,” “fun,” “interesting,” “glad I came”). Five youth participated in the one-on-one mentoring and several TRAs requested letters of reference for scholarship and college applications, including schools of nursing and medicine. TRA insights were critical to inform clinical trial protocols (NCT05509257, NCT04935931), recruitment strategies, and dissemination to scientific and lay communities via manuscripts and infographics (linktr.ee/StancilStudyTeam).

**Conclusion:**

We present a novel hybrid youth advisory board that reduces barriers to participation, fosters professional development, and substantially impacts the research program. Youth were highly engaged in online and in-person activities as well as collaboration synchronously and asynchronously. This model offers a scalable blueprint for engaging diverse adolescent populations in research, paving the way for more inclusive and impactful studies across disciplines.

## 1 Introduction

Engaging adolescents in research is paramount to ensure studies are relevant, ethical, beneficial, and that findings are authentic and applicable (1). Moreno et al. (2) highlights youth engagement [e.g., Youth Advisory Board, Youth Advisory Council, Teen Advisory Boards (TAB)] as a useful mechanism to influence study design, improve recruitment strategies, and ensure meaningful and practical interventions for youth. TABs leverage adolescent altruism while fostering ownership and trust (2–4).

Current descriptions of TABs in the literature describe in-person models that serve the whole institution or organization (5). Although advisory boards are employed across institutions, they remain underrepresented in the academic literature (2). Institution/organization-wide boards support *ad hoc* engagement with researchers for a specific question, grant application, etc., but do not generally support frequent, recurring interactions that inform ongoing operations of a specific research program. Traditional board members may or may not have lived experience with the condition being studied (e.g., study on eating disorders receiving input from youth who have not experienced an eating disorder) (6). Additionally, barriers to participation (e.g., transportation, scheduling constraints, and stigmatization of sensitive topics) are embedded in in-person models and may limit equitable access and inclusion of diverse perspectives (7). Innovative board structures that offer accessibility, flexibility, and closer alignment with the relevant research area are needed.

Sustaining adolescent engagement has traditionally been a challenge for research teams (3). Teens are more likely to stay engaged when their contributions are genuinely valued and they gain meaningful opportunities for growth (8). At the same time, researchers benefit from the perspective of youth with lived experience that improves the relevance, feasibility, and inclusivity of studies (3). This bidirectional exchange provides meaningful opportunities for adolescents while research programs are strengthened by authentic lived experience.

The primary aim of this manuscript is to describe the operational details of a novel, hybrid TAB that leverages online, asynchronous format and in-person educational and professional development experiences to engage youth with lived experience with the mental health conditions being studied, and as clinical trial participants. The secondary aim is to provide an evaluation of the initial impact of the program. We believe this TAB model has the potential for broader application in other adolescent research programs.

## 2 Methods

The Teen Research Advisors (TRA) is a community advisory board established in 2023 to involve youth and young adults with lived experience in clinical research. Program objectives were to: (1) engage and collaborate with youth to conduct adolescent research that is relevant, and (2) provide youth with professional development opportunities and mentorship to advance their career aspirations.

For program evaluation (see Section 2.4), we conducted secondary analysis of data collected during standard operations of the first 2 years of the TRA program (2023–2024). Data was collected from Parlay Ideas (e.g., discussion board posts and responses), meeting minutes (e.g., TRA roster, event attendance, and engagement duration), and elicited open-ended verbal feedback from staff and TRAs. This study was reviewed by the Institutional Review Board at Children’s Mercy Kansas City and was deemed exempt (non-human subjects research). De-identified data was analyzed using a mixed methods approach including thematic qualitative analysis of feedback from teens and staff and quantitative engagement metrics (Microsoft 365 Suite).

### 2.1 Formative group

**Figure 1:**
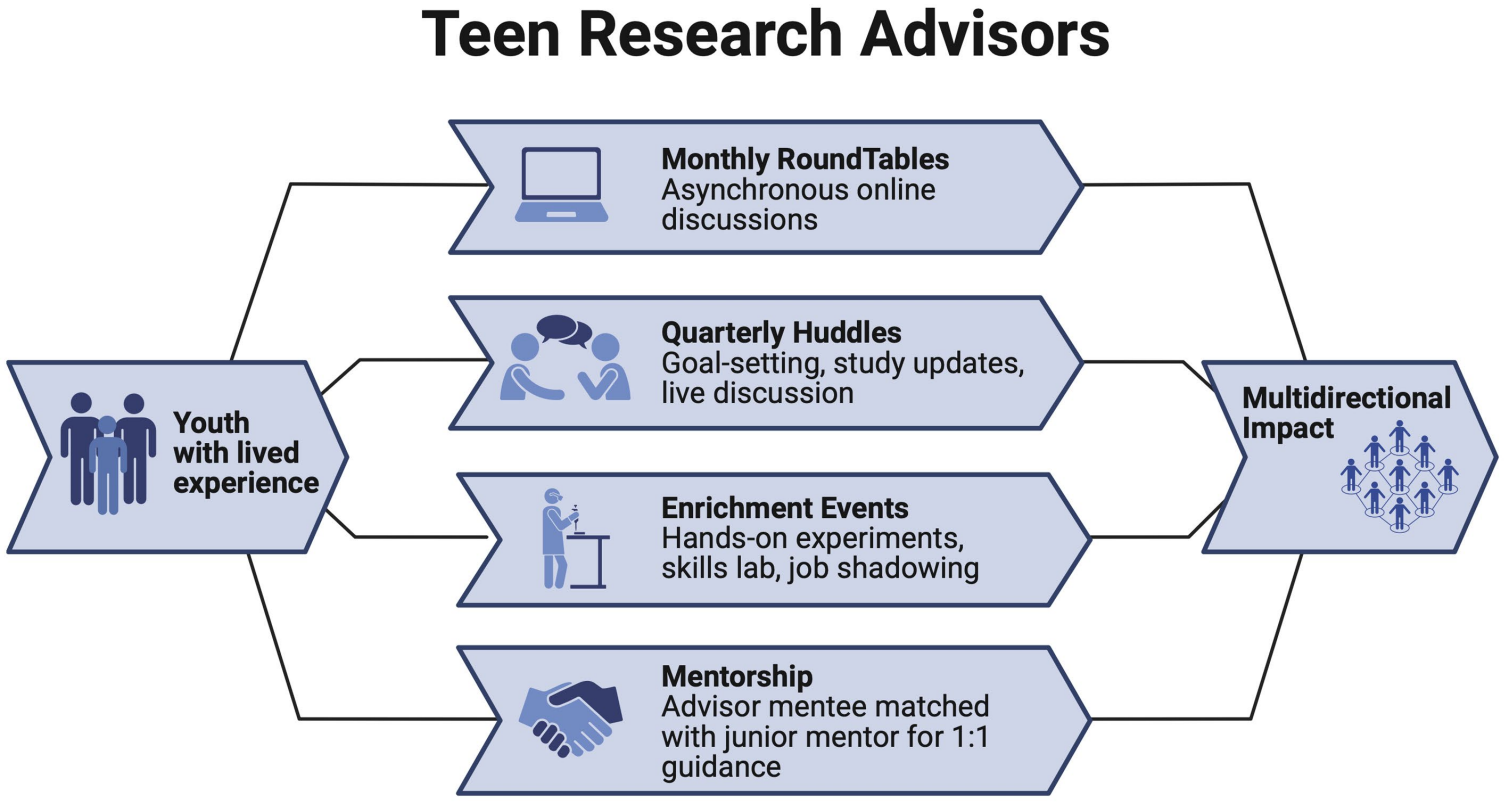
Structure of the teen research advisors program. Engagement though roundtables, huddles, enrichment events, and mentorship creates bidirectional impact by advancing research relevance while promoting youth professional development.

The development of the “Teen Research Advisors” consisted of a formative group of youth (*n* = 5) who developed the name, mission, goals, and initial structure of the TRA. Eligible teens were identified based on their past participation in a prior research study at Children’s Mercy Kansas City. The teens that expressed verbal interest in continuing involvement in research were contacted by email and invited to participate in the TAB. Informational letters were sent home to youth and parent/guardians. Participation included monthly discussions using an online asynchronous format to maximize participation at their own convenience (Parlay Ideas, see Section 2.3). The formative group was compensated quarterly ($50) for their time. Through ongoing collaboration over the first 6 months, Advisors directly shaped the priorities and activities of the TRA board (Figure 1).

### 2.2 Teen research advisors

Youth aged 13–21 years who had previously participated in a research study with our team at Children’s Mercy Kansas City (Kansas City, United States) were encouraged to apply. The TAB utilizes a continuous recruitment model allowing advisors to join or leave at any time. The goal is to continually recruit new Advisors as former Advisors move on from the program maintaining ~10 active Advisors at a time. Advisors receive a quarterly stipend of $50 and a certificate of participation. The program was developed using informed feedback from advisors to ensure it met their needs and preferences. The time commitment is approximately 4 h per quarter, including asynchronous participation in discussion boards, synchronous discussions, and events.

### 2.3 TRA activities

*Monthly discussion. Roundtables.* To enable communication that accommodated Advisors’ schedules and eliminated transportation barriers, an asynchronous discussion board format was piloted. Multiple online platforms were evaluated, prioritizing safety (e.g., closed groups) and optional Advisor anonymity. Parlay Ideas (Parlay Ideas Inc., Toronto, CA) was chosen for features that enhance discussion (e.g., comment prompts) and track metrics while meeting our safety and anonymity standards. Monthly discussion boards, called Roundtables, introduce a topic for Advisors to share responses under pseudonyms, promoting open dialogue and protecting privacy. In addition to posting their own response, Advisors were asked to respond to ≥1 other Advisor post. TRA Roundtable topics are workshopped within the lab team and informed by feedback/ideas gained during quarterly huddles. Topics alternated between areas of interest for Advisors (e.g., reducing mental health stigma, reading medical literature) and questions applicable to current research practices (e.g., study day procedures, recruitment process) and planning for future trials (e.g., trial design and implementation).

*Quarterly huddles*. Quarterly huddles were held synchronously (60 min sessions in-person and/or virtually) to set TRA priorities for the next 3–6 months (e.g., roundtable topics, enrichment events), share updates on studies, and offer a forum for live discussion and networking. Discussions topics were chosen by consensus among advisors and the research team.

*Enrichment events.* Enrichment events were offered twice a year (2 h in-person) to enhance engagement and provide professional development. The event focus is determined based on Advisors’ interests and may include hand-on laboratory experiments (e.g., DNA extraction, protein concentration determination), skills labs (e.g., how to measure vital signs), scientific publishing exposure (e.g., this manuscript), or professional shadowing in science and medicine.

*Mentorship*. A tiered mentorship approach was employed to provide meaningful career development for both Advisor mentee and their mentor. Advisors are paired with a junior mentor from the lab (JT, AB, MBa, MBr), who is a pre/professional student. Mentor-mentees are paired based on overlapping interests and career paths to foster compatibility and meaningful relationships. The mentor and mentee meet regularly (e.g., monthly) to cover topics such as goal setting, ‘work’/life balance, and upcoming applications. Junior mentors are mentored by the faculty member/principal investigator (SLS) who also provides primary oversight of the TRA program.

### 2.4 Initial program evaluation

We employed a mixed-methods descriptive approach to evaluate initial impact of the TRA program. Primary goal of evaluation was to ensure the program remained in alignment with the objectives defined by the Advisors (Advisor-defined objectives described in results). TRA-defined objectives were identified by consensus and iterated over time using monthly discussion boards, elicited feedback from TRAs monthly and at in-person events.

Program engagement was evaluated by reviewing extent and frequency of discussions during monthly Roundtables (Parlay Ideas, Inc.), with goal of ≥75% of Advisors posting an initial response. Responses to roundtables were summarized using inductive coding by lab members and shared at team meetings. Additionally, in person event participation rates and youth-reported perceptions of program relevance and professional development opportunities were elicited by open ended questions and analyzed by staff using an inductive approach to gauge program impact on TRA and used to inform future activities. Metrics (e.g., number of studies or study elements incorporating TRA insights) do not fully capture the extent of impact to the research program, thus qualitative thematic analysis augments the descriptive evaluation of research program impact.

TRA program description and initial evaluation was based on Standards for Reporting Implementation Studies (StaRI) guidelines (9).

## 3 Results

*Advisor Demographics.* During the first 2 years of the program (2023–2024), 11 youth aged 13–21 served as Advisors. The demographics of the TRA were consistent with the overall pool of those eligible to participate (Table 1).

**Table 1:** Demographics and engagement duration of teen research advisors.

Characteristics	Youth (*n* = 11)
Age, range (mean ± SD)	14–20 years (16.9 ± 2.1)
Gender identity*	86% Female
	14% Male
Race*	White: 82
	Asian: 9%
	Black or African American: 9%
Length of participation, range (mean ± SD)	4–24 months (12 ± 8)

*Age, race, and gender of the TRAs were consistent the pool of clinical research program participants during that time frame (*N* = 147, age range: 12–21 years; race: White 79%, Black 8%, Asian 4%, Other 9%; gender: 24% male, 70% female, 5% non-binary, 1% self-described).

The following sections describe qualitative TRA feedback and quantitative metrics organized into the TRA prioritized objectives, engagement and bidirectional impact.

### 3.1 Engagement

For any given monthly Roundtable (*n* = 23 Roundtables), >80% of TRAs contributed comments and peer responses. Quarterly Huddles (*n* = 7 huddles) were attended by 70% of TRAs and in-person enrichment events (*n* = 4 events) received positive feedback (“very helpful,” “fun,” “interesting,” “glad I came”). Youth with the highest levels of engagement particularly enjoyed the enrichment events and reported that seeing their suggestions put into action helped sustain their involvement. Several Advisors requested letters of reference for scholarship and college applications, including schools of nursing and medicine. Five youth participated in the mentoring with the majority engaging more than required by the program.

Advisors appreciated the structure of Parlay Ideas for its use of pseudonyms in protecting their identity while engaging in discussion with other members, a feature particularly beneficial to facilitating discussions of sensitive topics.

“I think that roundtables are a great way to answer prompts and be able to communicate and comment with other posts. I like how it’s somewhat anonymous and it’s easy to work with once you get used to it.”

Advisors shared that frequent interactions are important to maintain connectedness with the program. Our hybrid format allows regular participation on the advisors’ own time, supplemented by live discussions that foster a sense of community and provide in-depth exploration of ideas.

“… more peer discussion and communication [are] ideal to promote sharing ideas and perspectives. This could be achieved through more virtual meetings or live discussions.”

### 3.2 Bidirectional impact

*Impact on Teen Advisors.* Advisors perceived their contributions as ‘meaningful’ and ‘impactful’, prompting them to develop a sense of ownership with the board and research program. Advisors expressed positive feedback related to educational enrichment events and professional development experiences.

“The program empowers us to reach out and share our honest opinions surrounding the topics we care about. This extends to the encouragement of aspiring for additional leadership positions, professional experience, and individual ambitions in the medical field as well as personal life.”

“I think the sense of community draws one in. Some adolescents do not get to discuss topics like these with peers, nor do they get the opportunity to collaborate with healthcare professionals.”

“… this program impacts teenagers lives by giving them a platform to voice their opinions and experiences while also giving them opportunities to engage with research and learn more about healthcare.”

“This program can help me reach my goal by continuing to provide exposure to educational experiences in research and healthcare.”

**Figure 2:**
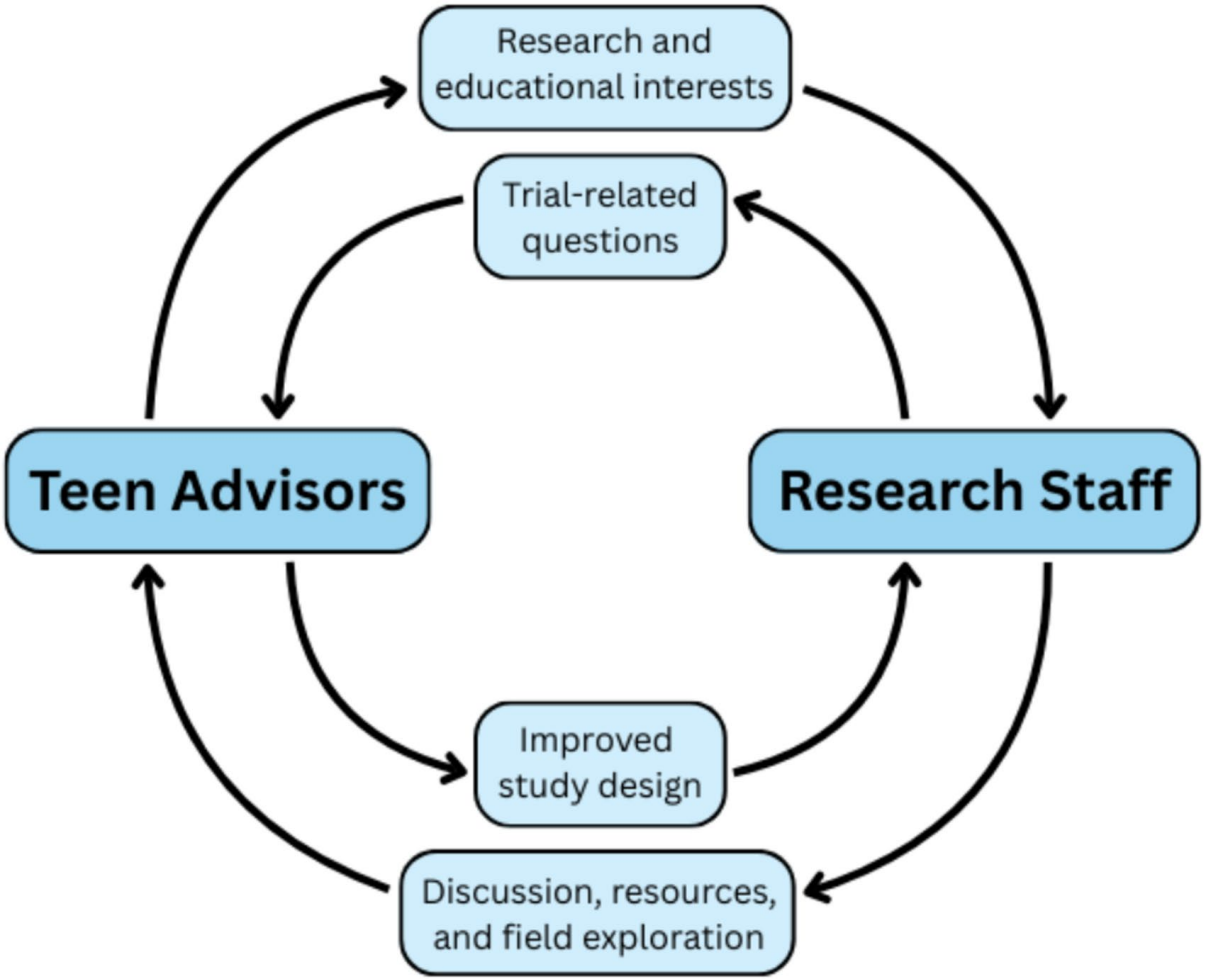
Impact of the teen research advisors program. Teen advisors contributed directly to multiple aspects of the research process, including clinical study protocol design, recruitment strategies, and dissemination of findings. Their lived experiences informed the development of participant-facing materials and improved study feasibility and retention. In addition to strengthening the research program, advisors benefited from mentorship, enrichment events, and professional development opportunities that supported their educational and career goals. This bidirectional model improves adolescent-focused research and fosters healthy youth development.

*Impact on Research Program.* Advisors collaborated on numerous aspects, through monthly Roundtables and live quarterly huddle discussions, including (1) clinical study protocol design [e.g., clinical trials: NCT05509257, NCT04935931; NIH grant proposals (PAR-25-180)], (2) community dissemination of research findings (e.g., infographics),^1^ (3) professional development of research team (e.g., junior mentoring opportunities), and (4) study operational improvement. Their lived experiences with mental health conditions and mental health research provided unique insights that strengthened study design, enhanced recruitment and retention strategies, and shaped dissemination efforts to be more accessible and relevant to their peers (Figure 2).

## 4 Discussion

We describe a novel hybrid Teen Research Advisors (TRA) program, intentionally designed to promote the inclusion of youth with lived experience while fostering bidirectional impact—ensuring mutual benefit for both youth participants and the research team. The program integrates advisory responsibilities with enrichment activities and mentorship, offering a well-rounded experience that acknowledges and builds upon the unique strengths of each individual. Notably, our TRA group functions independently of the institutional Teen Advisory Board and has specific goals (e.g., targeted inclusion of youth with lived experience of the conditions being studied, continuity with a primary research team) that are distinct from the institutional Teen Advisory Board, which generally focuses on *ad hoc* engagement across various research programs and investigators. Both programs provide youth in our community important opportunities to become engaged in research (10).

Leading professional bodies (e.g., Society for Adolescent Health and Medicine) urge researchers to involve adolescents and their communities in study design and priority-setting (11). Indeed, because of the continuity with the research team, our TRAs contribute to all aspects of the clinical translational research program focused on adolescent precision therapeutics and their insights, captured during monthly Roundtables and quarterly huddles, enhance the relevance and feasibility of study protocols, improved participant-facing materials, and shaped dissemination strategies to be more accessible to adolescent audiences. In recognition of their substantial contributions, several Teen Advisors were included as co-authors on this manuscript, and all were given the opportunity. Beyond contributing to community-engaged research objectives, our TRA model also prioritizes youth personal and professional development.

Adolescence is a key period for developing autonomy and identity (12). Giving youth meaningful roles in the community can help to build their sense of purpose (13, 14). Opportunities that promote autonomy and altruism such as sustained mentorship and community engagement are developmentally aligned with adolescents’ needs for identity formation (15). These experiences support community contribution and play a critical role in helping youth transition to adulthood. The TRA model supports the personal and professional development of Teen Advisors. For youth, participation provides exposure to clinical research, hands-on experiences in science and medicine, and mentorship tailored to their interests. Mentorship improves physical health, mental health and career outcomes for youth (16, 17). The sustained mentorship offered through the program centered on goal setting and helping teens identify opportunities that aligned with their personal and professional aspirations. Additionally, junior research team members gained valuable experience in mentorship and community engagement which supported their own professional growth. This mutual exchange of knowledge and growth underscores the value of adolescent partnership in research as both a scientific and educational investment.

The hybrid format, including online synchronous, asynchronous, and in-person opportunities, has been successful in generating a sense of ownership resulting in high TRA engagement rates in all activities. Advisors were engaged as active collaborators rather than passive participants. The flexibility of this structure addressed common barriers (e.g., transportation, scheduling conflicts, participation anxiety) to participation and allowed youth to contribute in ways that best fit their schedules and comfort levels (7, 18). While attended by most TRAs, in person events did suffer from these participation barriers. We found offering hybrid events in addition to in person enrichment experiences was important to our TRAs to optimize opportunities for participation. This approach aligns with prior work that showed that flexible participation models increased accessibility and equity of participation for youth (19). The use of Parlay Ideas as the asynchronous discussion platform further enhanced usability and engagement by offering a secure, youth-friendly interface that supported anonymity and encouraged peer-to-peer interaction. These features fostered open dialogue and created a forum that ensures that all voices were heard. As a result of the program design, our teen advisors rapidly became valued members of the research team, contributing to grant submissions and the development of study day procedures.

TRA program evaluation utilized a mixed methods approach incorporating regular assessment of engagement (detailed in Methods) and qualitative feedback from Advisors and research staff. Existing reports of youth research advisory programs focus on program description and often rely on limited metrics, with most focusing primarily on tracking in-person attendance (5). Sustained involvement and stories of personal growth and satisfaction from both TRAs and our study team members further affirm the value of the program. The integration of youth perspectives into the core research processes fostered a more inclusive, reflective, and responsive research culture within the team. Our initial program evaluation may have involved bias such as social desirability bias (TRAs sharing what they believe study staff wanted to hear) and selection bias (as participants were previously engaged in research). However, TRAs did share constructive criticism that allowed iteration of program elements. A future formal evaluation of the program by an external investigator may reduce bias and augment understanding of impact but was outside the scope of the current work.

We acknowledge that programs may exist that integrate similar features to those described in this paper; however, to our knowledge this is the first formal description of a youth advisory board comprised of youth with lived experience with the conditions being studied, using a hybrid format (e.g., in person and virtual, synchronous and asynchronous), and prioritizing youth professional development for future careers in medicine and science. Unlike many advisory boards that operate in-person only or provide support for multiple research/clinical programs (e.g., the University of Alabama at Birmingham’s Youth Advisory Board) our Teen Research Advisors (TRA) program is embedded into the long-term operations of a clinical research lab and supports ongoing, youth-driven agenda setting. Additionally, our use of an anonymous online discussion platform (Parlay Ideas) for monthly roundtables is a unique strategy to facilitate open and equitable participation in sensitive health topics. The operational details and description of initial impact aim to support adoption of teen advisory boards across a broad range of clinical research programs.

The future of the TRA program continues to be determined through ongoing Advisor engagement to ensure our research remains closely aligned with the needs and preferences of the youth it strives to serve. As the program evolves, we aim to continue integrating Advisor feedback into both strategic planning and day-to-day research operations. For other research teams seeking to implement similar models, key next steps include identifying youth with lived experience relevant to the research focus, establishing flexible engagement formats that reduce participation barriers, and building sustainable mentorship structures. Development should be iterative and co-designed with youth input from the outset to foster trust and ownership. Implementation should prioritize accessibility and continuous evaluation to adapt the model to diverse research settings. By adopting and customizing elements of the TRA model, research teams can advance more inclusive, relevant, and impactful adolescent health research.

## Data Availability

The raw data supporting the conclusions of this article will be made available by the authors, without undue reservation, upon relevant regulatory and ethics approval.

## Footnotes

^1^ https://linktr.ee/StancilStudyTeam
